# Real-world evidence of characteristics and factors influencing herbal medicine use for weight loss in adults

**DOI:** 10.3389/fphar.2024.1437032

**Published:** 2024-07-16

**Authors:** Boram Lee, Changsop Yang, Mi Hong Yim

**Affiliations:** ^1^ KM Science Research Division, Korea Institute of Oriental Medicine, Daejeon, Republic of Korea; ^2^ Digital Health Research Division, Korea Institute of Oriental Medicine, Daejeon, Republic of Korea

**Keywords:** herbal medicine, obesity, overweight, weight loss, korea national health nutrition examination survey

## Abstract

**Background:**

Obesity imposes a significant socioeconomic burden owing to its high prevalence. In response to the adverse outcomes associated with conventional pharmacotherapy and the challenges of low adherence to lifestyle interventions, herbal medicine has surfaced as an actively utilized approach for weight loss. Therefore, this study aimed to analyze the characteristics and influencing factors of herbal medicine users for weight loss in adults.

**Methods:**

Overall, 22,080 participants were included based on data from the Korea National Health and Nutrition Examination Survey from 2010 to 2019. Simple logistic regression analyses were used to derive the associations between herbal medicine use for weight loss and individual characteristics. Three models were constructed utilizing multiple logistic regression analyses to assess the associations between herbal medicine use for weight loss and the combined characteristics of predisposing, enabling, and need factors according to Andersen’s model.

**Results:**

In the full adjustment model, women, younger adults, those with higher incomes, and individuals reporting higher levels of perceived stress were more prone to use herbal medicine for weight loss in the past year. Adults who identified body image as being fat/very fat, those who consumed alcohol, and those classified as severely obese by body mass index were also more prone to use herbal medicine for weight loss. In particular, adults with a higher rate and amount of weight loss in the past year were more likely to use herbal medicine for weight loss compared to those experiencing weight gain/no changes/loss of 0–3 kg.

**Conclusion:**

Our study was the first to derive the characteristics and influencing factors of herbal medicine users for weight loss among adults. These findings hold significant promise for informing future research endeavors and policy decision-making for effective resource distribution for obesity treatment.

## 1 Introduction

Over the past 40 years since 1975, the global prevalence of obesity has nearly tripled, causing a significant socioeconomic burden worldwide, such as increased healthcare costs and loss of productivity (Tremmel et al., 2017; World Health Organization, 2021; Malkin et al., 2022). Preventing obesity is significant from a public health perspective for controlling non-communicable diseases such as metabolic syndrome, cardiovascular diseases, and cancers (Henry, 2011; Tiwari and Balasundaram, 2023). Additionally, beyond health concerns, a significant increase has been observed in the desire for weight loss, motivated by aesthetic reasons, irrespective of an individual body mass index (BMI), in recent years (Boepple et al., 2019; Teng et al., 2020). Data indicates that approximately 50% of US adults have attempted to lose weight, of which 66.7% were obese, while 26.5% were either underweight or normal weight (Martin et al., 2018). This underscores a significant interest in treating and preventing obesity for both health and aesthetic reasons.

Conventional treatments for obesity include lifestyle-related therapy, pharmacotherapy, and surgical procedures (Kim et al., 2023). However, owing to the potential for adverse effects associated with pharmacotherapy and poor adherence to lifestyle intervention (Lewis, 2019; Tak and Lee, 2021), herbal medicine (HM) has been used as an alternative for weight loss purposes (Eldalo et al., 2017; Cheon and Jang, 2021). In South Korea, Korean medicine, including HM, is recognized alongside conventional medicine. Moreover, a Korean medicine registry focusing on HM for weight loss has been established to investigate the clinical characteristics and safety profiles of various HM treatments (Ko et al., 2022). In addition, several clinical studies have been conducted to evaluate the effectiveness and safety of HM in managing obesity (Maunder et al., 2020; Park et al., 2022). According to the recent network meta-analysis, HM and HM plus acupuncture significantly reduced BMI compared with lifestyle management in children with simple obesity (Lee and Kwon, 2022). Furthermore, HM combined with acupuncture showed significantly higher total effective rate based on the improvement of obesity and related symptoms when compared with fenfluramine (Lee and Kwon, 2022).

Recently, policymaking according to evidence-based, real-world data (RWD) is increasing (Cave et al., 2019; Kc et al., 2023), with RWD finding utility in the integrative medicine field for scrutinizing health outcomes, healthcare utilization, and associated factors (Lee et al., 2022a; Lee et al., 2022b; Lee et al., 2023a; Lee et al., 2023b). By analyzing the characteristics and factors of HM users for weight loss, the demographics and variables of individuals who have unmet medical needs within conventional obesity treatment were identified. Addressing this population is significant as it mitigates serious socioeconomic risks. This information can be utilized as foundational data for establishing policies for efficient resource allocation. Additionally, the identified traits and factors can serve as valuable inputs when devising analysis methodologies for future prospective studies in this field. Factors influencing the use of HM for weight loss in children and adolescents have been recently reported (Yim and Lee, 2023); however, to our knowledge, no relevant studies have been conducted focusing on adults. Therefore, this study aimed to investigate the characteristics and influencing factors of adults using HM for weight loss. We utilized the Korea National Health and Nutrition Examination Survey (KNHANES) as our primary data source, which provides representative RWD.

## 2 Methods

### 2.1 Data source and study participants

In this study, data from the fifth (2010–2012), sixth (2013–2015), seventh (2016–2018), and eighth (2019) KNHANES were used. KNHANES, implemented nationwide by the Korea Disease Control and Prevention Agency since 1998, serves as a fundamental data source for health policy development and evaluation, including national health promotion programs. The survey comprises a health interview conducted either a face-to-face interview or via self-administered questionnaires at mobile examination centers, health examinations involving measurements or assessments at mobile examination centers, and a nutrition survey conducted through face-to-face interviews at homes of individuals that were sampled (Kim, 2014; Kweon et al., 2014). Utilizing a complex sampling design, KNHANES ensures the representativeness and reliability of data collected from the nationwide target population (Kweon et al., 2014). Data from KNHANES are provided in a de-identified format, ensuring individual confidentiality, and can be accessed and downloaded from the official KNHANES website (https://knhanes.kdca.go.kr/knhanes/main.do). In this study, we analyzed health interview and examination data from KNHANES from 2010 to 2019 to identify the characteristics and factors associated with HM users for weight loss among adults. Consequently, the institutional review board of the Korea Institute of Oriental Medicine waived the requirement for ethics approval for this study (IRB No., I-2312/012-005).

**FIGURE 1:**
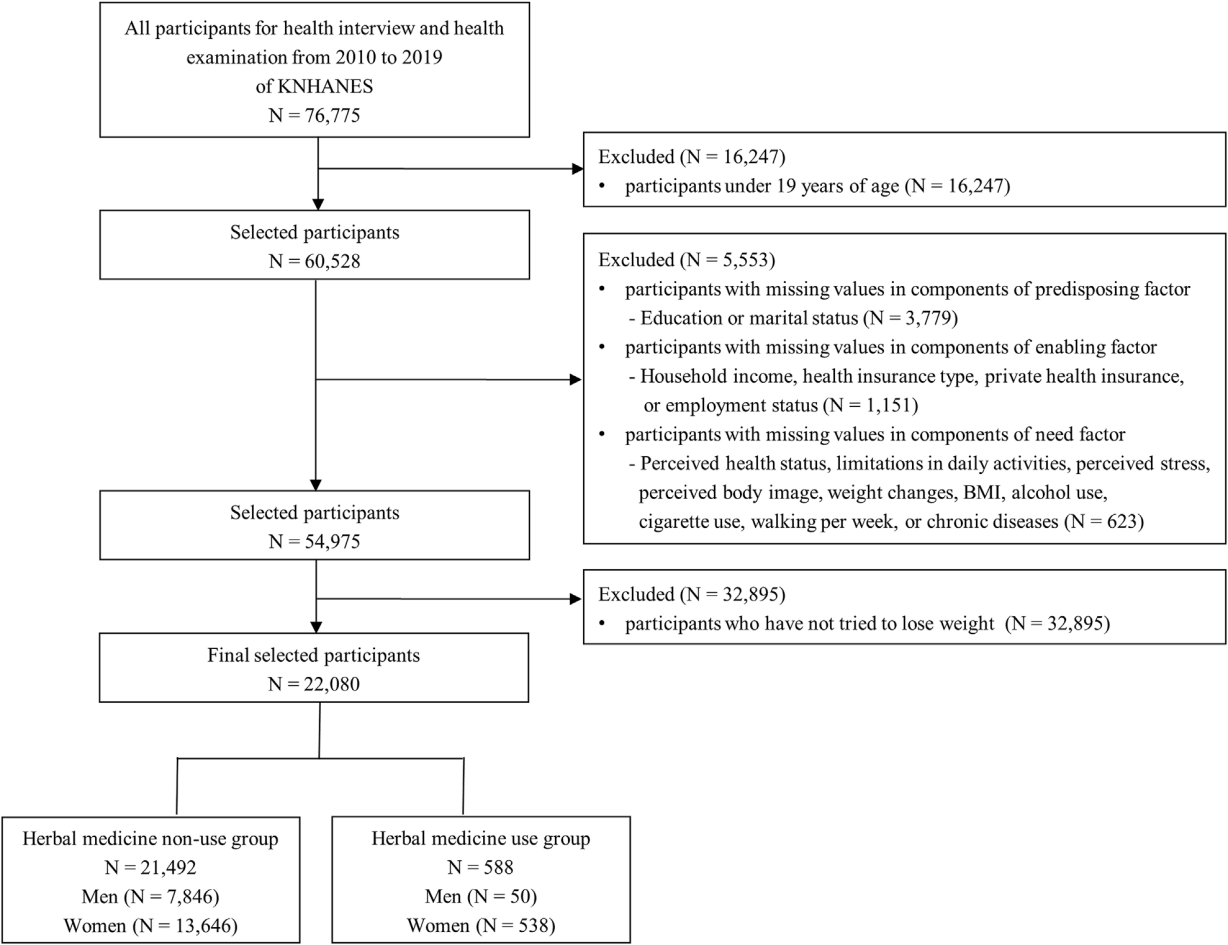
Flowchart of the selection process of study participants.

Overall, 76,775 individuals participated in health interviews and examinations of KNHANES from 2010 to 2019. We chose 60,528 participants, excluding 16,247 participants under 19 years of age. After further exclusion of 5,223 participants with missing survey data, 54,975 participants were selected. Of these, 32,895 participants who had not attempted weight loss were excluded. Finally, 22,080 (7,896 men and 14,184 women) participants, including data from 588 HM users (50 men and 538 women) and 21,492 non-users (7,846 men and 13,646 women) were analyzed (Figure 1).

### 2.2 Definition of herbal medicine used for weight loss

The HM users for weight loss were identified based on their responses to two questions: “Have you attempted weight control on your own in the past year?” and “What methods have you utilized for weight loss in the past year?” Participants who responded affirmatively to both questions, indicating they had attempted weight loss and had used HM, were categorized as the HM user group. Conversely, participants who indicated they had attempted weight loss but did not report HM use were classified as the HM non-user group. Responses excluding HM use for the second question included exercise, fasting for more than 24 h, eating a little, skipping meals, over-the-counter weight loss medicine, prescribed weight loss medication, healthy functional foods, and a monotrophic diet. Responses of participants to both questions were collected through self-administered surveys, adhering to guidelines that included detailed explanations for each question. This approach aimed to ensure participants understood the questions clearly and to minimize any potential recall bias (Korea Disease Control and Prevention Agency, 2012b; Korea Disease Control and Prevention Agency, 2013; Korea Disease Control and Prevention Agency, 2014; Korea Disease Control and Prevention Agency, 2015b; Korea Disease Control and Prevention Agency, 2016; Korea Disease Control and Prevention Agency, 2017; Korea Disease Control and Prevention Agency, 2018b; Korea Disease Control and Prevention Agency, 2019b).

### 2.3 Measures

Three factors, including individual characteristics, were analyzed based on Andersen’s theoretical framework to identify the characteristics and influencing factors of adult HM users for weight loss (Andersen, 1995; Babitsch et al., 2012; Von Lengerke et al., 2013). Andersen’s behavioral model provides a theoretical framework for understanding and predicting access to and utilization of healthcare services and identifying factors that influence the health behaviors and decisions of an individual (Andersen, 1995; Andersen, 2008). According to this model, healthcare service utilization is influenced by predisposing, enabling, and need factors, which respectively refer to the demographic characteristics of an individual, ability to access healthcare services, and motivation to utilize them (Andersen, 1995; Babitsch et al., 2012; Von Lengerke et al., 2013). In this study, predisposing factors identified from KNHANES questions included sex, age, region, education, and marital status, while enabling factors consisted of residential areas, household income, number of household members, employment status, health insurance type, and private health insurance. Need factors included perceived health status, limitations in daily activities, perceived stress, depression, perceived body image, weight changes, BMI, alcohol use, cigarette use, weekly walking frequency, hypertension, dyslipidemia, diabetes mellitus, and other chronic disease. Specifically, depression was categorized as either present or absent based on whether individuals had received a current diagnosis of depression from a physician. Weight changes were classified according to the amount of weight lost over the past year. Alcohol use was categorized based on the consumption rate in the past year, cigarette use was categorized based on the current smoking habit of an individual, and walking per week was categorized based on the number of days individuals walked for more than 10 min at a time during the past week. Hypertension, dyslipidemia, and diabetes mellitus were categorized based on the current presence or absence of each disease after diagnosis by a physician. Other chronic disease was determined based on the presence or absence of a diagnosed disease among the following nine conditions: stroke, myocardial infarction or angina pectoris, arthritis, pulmonary tuberculosis, asthma, atopic dermatitis, thyroid disease, cancer, and chronic liver disease (hepatitis B, hepatitis C, or hepatic cirrhosis). All characteristics, except for BMI, were obtained from health interviews. BMI was calculated using height and weight measurements obtained during health examinations performed at mobile examination centers, using calibrated equipment operated by well-trained medical staff following standardized protocols (Korea Disease Control and Prevention Agency, 2012a; Korea Disease Control and Prevention Agency, 2015a; Korea Disease Control and Prevention Agency, 2018a; Korea Disease Control and Prevention Agency, 2019a).

### 2.4 Statistical analysis

The KNHANES data were collected using a two-stage stratified cluster sampling method rather than simple random sampling to ensure a representative sample of the nationwide target population. Consequently, the probability of being selected as a sample is not uniform across all population members. Sample weights were used for all statistical analyses to adjust for unequal selection probabilities and nonresponse errors. As data from the fifth to eighth wave of the KNHANES were combined and used in this study, weighted analyses were conducted using the sample weights modified according to the guidelines of the KNHANES.

To compare individual characteristics between HM users and non-users, chi-squared tests with Rao–Scott correction were applied because all individual characteristics were categorical variables. The results were presented as the frequency and weighted column percentage. Simple logistic regression analyses were used to assess the associations between HM use for weight loss and individual characteristics related to predisposing, enabling, and need factors. The results were summarized as crude odds ratios (cORs) with their corresponding 95% confidence intervals (95% CIs). Additionally, three models were constructed using multiple logistic regression analyses to examine the associations between HM use for weight loss and combined characteristics of predisposing, enabling, and need factors. Model one included the combined characteristics of predisposing factors, model two consisted of the combined characteristics of predisposing and enabling factors, and model three comprised the combined characteristics of predisposing, enabling, and need factors. The results were summarized as adjusted odds ratios (aORs) with their corresponding 95% CIs. To assess the fit of logistic regression, the pseudo R-square of Nagelkerke was determined (Nagelkerke, 1991). All statistical analyses were performed using R version 4.2.1 (R Foundation for Statistical Computing, Vienna, Austria) (Team RC. R, 2023) and IBM SPSS Statistics for Windows, version 29.0 (IBM Corp., Armonk, NY, United States) (Corp, 2022). A significant level of 0.05 and a two-tailed test were used for all statistical analyses.

## 3 Results

### 3.1 Characteristics of herbal medicine user for weight loss in adults

Significant differences were observed between HM users and non-users in several demographic and health-related factors, including sex, age, region, education, household income, number of household members, health insurance type, private health insurance, perceived stress, perceived body image, weight changes, BMI, alcohol use, cigarette use, hypertension, dyslipidemia, and diabetes mellitus. The proportion of women among HM users (89.4%) was significantly higher compared to non-users (57.49%). Other characteristics that demonstrated higher proportions among the HM users compared to the non-users include younger age, higher education level, higher house income, subscription to private health insurance, higher level of perceived stress, the higher percentage reporting a perceived body image as “very fat,” greater weight loss in the past year, higher BMI, lower cigarette use, and absence of hypertension, dyslipidemia, or diabetes mellitus (Table 1).

**TABLE 1:** General characteristics of participants between herbal medicine users and non-users.

Variables	Men and women			Men		Women	
	Herbal medicine			Herbal medicine		Herbal medicine	
	Non-use	Use	*p*-value	Non-use	Use	Non-use	Use
Number of participants	21,492	588		7,846	50	13,646	538
Predisposing factors							
Sex			<.001				
Men	7,846 (42.51)	50 (10.6)					
Women	13,646 (57.49)	538 (89.4)					
Age (years)			<.001				
65 or older	3,407 (9.25)	21 (2.19)		1,378 (8.71)	6 (7.01)	2029 (9.66)	15 (1.62)
50-64	6,376 (26.34)	112 (14.86)		2,278 (26.1)	5 (9.65)	4,098 (26.51)	107 (15.48)
35-49	6,935 (34.06)	257 (40.32)		2,503 (34.42)	22 (40.96)	4,432 (33.79)	235 (40.24)
19-34	4,774 (30.35)	198 (42.63)		1,687 (30.77)	17 (42.37)	3,087 (30.04)	181 (42.66)
Region			.005				
Seoul/Gyeonggi/Incheon	11,209 (52.56)	339 (59.72)		4,130 (52.85)	30 (68.43)	7,079 (52.34)	309 (58.69)
Gangwon	694 (2.98)	18 (2.02)		287 (3.29)	1 (0.44)	407 (2.75)	17 (2.2)
Daejeon/Chungcheong/Sejong	2,223 (10.27)	66 (11.27)		827 (10.65)	6 (9.36)	1,396 (10)	60 (11.49)
Gwangju/Jeolla/Jeju	2,438 (10.15)	49 (6.22)		890 (9.58)	3 (3.84)	1,548 (10.57)	46 (6.5)
Busan/Daegu/Ulsan/Gyeongsang	4,928 (24.04)	116 (20.77)		1712 (23.63)	10 (17.93)	3,216 (24.35)	106 (21.11)
Education			<.001				
Elementary school or below	3,391 (11.16)	36 (5.27)		788 (6.7)	3 (7.26)	2,603 (14.46)	33 (5.04)
Middle	2,101 (8.36)	31 (5.22)		713 (7.2)	0 (0)	1,388 (9.21)	31 (5.84)
High school	7,696 (38.7)	236 (39.9)		2,772 (38.1)	20 (32.66)	4,924 (39.14)	216 (40.76)
College or above	8,304 (41.78)	285 (49.6)		3,573 (48)	27 (60.08)	4,731 (37.19)	258 (48.36)
Marital status			.106				
Married/living together	15,469 (67.53)	426 (67.89)		5,891 (67.68)	33 (59.82)	9,578 (67.41)	393 (68.85)
Widowed/Divorced/Separated	2,277 (8.29)	40 (5.6)		368 (3.81)	(0)	1909 (11.61)	40 (6.27)
Never married	3,746 (24.18)	122 (26.5)		1,587 (28.51)	17 (40.18)	2,159 (20.98)	105 (24.88)
Enabling factors							
Residential areas			.095				
Urban	18,023 (85.36)	516 (88.48)		6,549 (85.15)	46 (93.44)	11,474 (85.51)	470 (87.9)
Rural	3,469 (14.64)	72 (11.52)		1,297 (14.85)	4 (6.56)	2,172 (14.49)	68 (12.1)
Household income			<.001				
1st quintile(lowest)	3,007 (11.87)	33 (5.23)		981 (10.44)	1 (0.52)	2026 (12.93)	32 (5.79)
2nd quintile	3,937 (17.79)	87 (14.99)		1,301 (15.85)	7 (14.02)	2,636 (19.22)	80 (15.1)
3rd quintile	4,513 (21.9)	124 (21.76)		1,625 (21.51)	5 (13.53)	2,888 (22.19)	119 (22.73)
4th quintile	4,817 (23.57)	155 (29.02)		1839 (24.77)	15 (28.94)	2,978 (22.69)	140 (29.03)
5th quintile (highest)	5,218 (24.86)	189 (29.01)		2,100 (27.43)	22 (42.99)	3,118 (22.96)	167 (27.35)
Number of household members			.001				
1 member	1786 (7.23)	18 (3.39)		583 (7.41)	3 (6.45)	1,203 (7.09)	15 (3.02)
2 members	5,493 (21.19)	110 (16.57)		2068 (20.61)	9 (14.8)	3,425 (21.61)	101 (16.78)
3 members	5,579 (27.85)	163 (28.64)		2045 (27.8)	13 (34.64)	3,534 (27.88)	150 (27.93)
4 members	6,230 (31.76)	220 (38.84)		2,337 (32.88)	18 (29.78)	3,893 (30.93)	202 (39.92)
≥5 members	2,404 (11.98)	77 (12.56)		813 (11.29)	7 (14.33)	1,591 (12.48)	70 (12.35)
Employment status			.266				
Employed	9,161 (46.79)	267 (47.72)		4,039 (55.18)	30 (64.23)	5,122 (40.59)	237 (45.76)
Self-employed	3,293 (15.38)	77 (12.52)		1887 (22.65)	12 (23.05)	1,406 (10)	65 (11.27)
Unpaid family worker/unemployed	9,038 (37.83)	244 (39.76)		1920 (22.17)	8 (12.71)	7,118 (49.41)	236 (42.97)
Health insurance type			.033				
Local-subscriber	6,692 (31.93)	165 (29.09)		2,423 (31.45)	13 (24.28)	4,269 (32.29)	152 (29.66)
Employee health insurance	14,252 (65.66)	418 (70.01)		5,256 (66.53)	37 (75.72)	8,996 (65.02)	381 (69.33)
Medical aid or others	548 (2.41)	5 (0.9)		167 (2.03)	(0)	381 (2.69)	5 (1.01)
Private health insurance			<.001				
Yes	17,708 (84.89)	545 (92.69)		6,316 (84.17)	45 (89.95)	11,392 (85.42)	500 (93.01)
No	3,784 (15.11)	43 (7.31)		1,530 (15.83)	5 (10.05)	2,254 (14.58)	38 (6.99)
Need Factors							
Perceived health status			.575				
Very good/Good	6,880 (33.3)	187 (31.61)		2,820 (37.22)	10 (21.81)	4,060 (30.4)	177 (32.78)
Fair	10,822 (50.41)	299 (50.37)		3,882 (49.06)	27 (51)	6,940 (51.4)	272 (50.3)
Poor/Very poor	3,790 (16.29)	102 (18.02)		1,144 (13.71)	13 (27.2)	2,646 (18.2)	89 (16.93)
Limitations in daily activities			.581				
Yes	1,651 (6.56)	34 (5.83)		552 (6.18)	4 (5.65)	1,099 (6.83)	30 (5.85)
No	19,841 (93.44)	554 (94.17)		7,294 (93.82)	46 (94.35)	12,547 (93.17)	508 (94.15)
Perceived stress			<.001				
Barely	2,964 (12.36)	52 (8.02)		1,208 (13.29)	6 (7.96)	1756 (11.68)	46 (8.03)
Low	12,636 (58.66)	303 (49.63)		4,673 (59.61)	17 (30.44)	7,963 (57.95)	286 (51.9)
High	4,874 (24.02)	186 (32.11)		1,659 (23.06)	23 (50.44)	3,215 (24.72)	163 (29.94)
Very high	1,018 (4.96)	47 (10.24)		306 (4.04)	4 (11.15)	712 (5.64)	43 (10.13)
Depression			.151				
No	20,945 (97.72)	566 (96.65)		7,743 (98.72)	49 (99.07)	13,202 (96.98)	517 (96.36)
Yes	547 (2.28)	22 (3.35)		103 (1.28)	1 (0.93)	444 (3.02)	21 (3.64)
Perceived body image			<.001				
Very thin/thin/moderate	7,214 (34.01)	92 (15.01)		2,486 (31.16)	5 (10.87)	4,728 (36.12)	87 (15.5)
Fat	11,246 (51.94)	325 (54.42)		4,499 (57.14)	23 (44.69)	6,747 (48.08)	302 (55.57)
Very fat	3,032 (14.05)	171 (30.57)		861 (11.69)	22 (44.44)	2,171 (15.8)	149 (28.92)
Weight changes			<.001				
Gain/No changes/Loss of 0–3 kg	18,025 (82.85)	427 (71.27)		6,342 (80.1)	33 (68.09)	11,683 (84.88)	394 (71.65)
Loss of 3–6 kg	2,519 (11.92)	84 (14.38)		1,044 (13)	6 (9.97)	1,475 (11.12)	78 (14.9)
Loss of 6–10 kg	639 (3.4)	55 (10.16)		314 (4.59)	7 (13.27)	325 (2.52)	48 (9.8)
Loss of 10 kg or more	309 (1.84)	22 (4.19)		146 (2.31)	4 (8.67)	163 (1.48)	18 (3.66)
BMI (kg/m^2^)			<.001				
<23	5,734 (27.4)	152 (26.37)		747 (10.33)	1 (0.98)	4,987 (40.02)	151 (29.38)
23.0-24.9	5,170 (23.92)	135 (20.86)		2026 (25.77)	6 (14.69)	3,144 (22.55)	129 (21.59)
25.0-29.9	8,981 (40.94)	210 (35.16)		4,424 (54.92)	22 (40.42)	4,557 (30.61)	188 (34.53)
≥30	1,607 (7.74)	91 (17.62)		649 (8.99)	21 (43.91)	958 (6.82)	70 (14.5)
Alcohol use			.009				
None	5,244 (20.66)	111 (15.36)		1,170 (12.84)	3 (6.51)	4,074 (26.44)	108 (16.41)
Monthly or less	6,675 (30.69)	219 (37.83)		1,573 (20.96)	13 (26.89)	5,102 (37.88)	206 (39.13)
2 to 4 times a month	5,225 (26.64)	143 (26.57)		2,349 (31.7)	14 (30.87)	2,876 (22.89)	129 (26.06)
2 to 3 times a week	3,240 (16.7)	87 (15.05)		1965 (25.46)	14 (27.65)	1,275 (10.22)	73 (13.55)
4 or more times a week	1,108 (5.31)	28 (5.2)		789 (9.03)	6 (8.08)	319 (2.57)	22 (4.85)
Cigarette use			<.001				
None/Quit smoking	18,195 (81.06)	527 (88.15)		5,300 (64.6)	30 (61.15)	12,895 (93.23)	497 (91.35)
Occasionally	560 (3.2)	19 (3.68)		343 (4.83)	3 (6.03)	217 (1.99)	16 (3.4)
Every day	2,737 (15.74)	42 (8.17)		2,203 (30.57)	17 (32.82)	534 (4.78)	25 (5.24)
Walking per week			.576				
None	2,934 (12.83)	67 (11.32)		1,028 (12.87)	12 (23.49)	1906 (12.8)	55 (9.87)
1–2 days	3,649 (16.95)	108 (17.78)		1,323 (16.61)	4 (9.77)	2,326 (17.21)	104 (18.73)
3–4 days	4,751 (21.4)	135 (23.01)		1,657 (20.31)	10 (18.27)	3,094 (22.21)	125 (23.58)
5–6 days	3,941 (18.91)	118 (20.52)		1,392 (18.11)	8 (17.88)	2,549 (19.51)	110 (20.84)
Every day	6,217 (29.9)	160 (27.37)		2,446 (32.11)	16 (30.59)	3,771 (28.26)	144 (26.98)
Hypertension			<.001				
No	16,845 (83.23)	529 (92.19)		5,824 (80.79)	39 (80.96)	11,021 (85.04)	490 (93.52)
Yes	4,647 (16.77)	59 (7.81)		2022 (19.21)	11 (19.04)	2,625 (14.96)	48 (6.48)
Dyslipidemia			.002				
No	18,687 (89.67)	542 (93.76)		6,844 (89.76)	42 (87.26)	11,843 (89.6)	500 (94.53)
Yes	2,805 (10.33)	46 (6.24)		1,002 (10.24)	8 (12.74)	1803 (10.4)	38 (5.47)
Diabetes mellitus			.006				
No	19,817 (93.96)	568 (96.9)		7,048 (92.73)	45 (90.15)	12,769 (94.88)	523 (97.69)
Yes	1,675 (6.04)	20 (3.1)		798 (7.27)	5 (9.85)	877 (5.12)	15 (2.31)
Other chronic disease			.134				
No	16,983 (83.06)	499 (85.79)		6,657 (88)	43 (88.48)	10,326 (79.41)	456 (85.47)
Yes	4,509 (16.94)	89 (14.21)		1,189 (12)	7 (11.52)	3,320 (20.59)	82 (14.53)

Abbreviations: BMI, body mass index.

*p* values were derived from chi-squared tests with Rao-Scott correction for the categorical variables. The results were summarized as the frequency and weighted column percentage.

### 3.2 Influencing factors of herbal medicine user for weight loss in adults

In the initial analyses, significant associations were observed between HM use for weight loss and individual characteristics, including sex, age, region, education, household income, number of house members, private health insurance, perceived stress, perceived body image, weight changes, BMI, alcohol use, cigarette use, hypertension, dyslipidemia, and diabetes mellitus. However, some of these associations lost significance after adjusting sequentially for predisposing, enabling, and need factors. In model 1, which considered predisposing factors, significant associations were found between HM use for weight loss and sex, age, and region. Model 2, incorporating both predisposing and enabling factors, revealed significant associations between HM use for weight loss and sex, age, region, and household income. Model 3, including predisposing, enabling, and need factors, showed significant associations between HM use for weight loss and the following characteristics: sex, age, region, household income, perceived stress, perceived body image, weight changes, BMI, and alcohol use. The pseudo R-square values of Nagelkerke were 0.089, 0.096, and 0.17 for model 1, model 2, and model 3, respectively.

**FIGURE 2:**
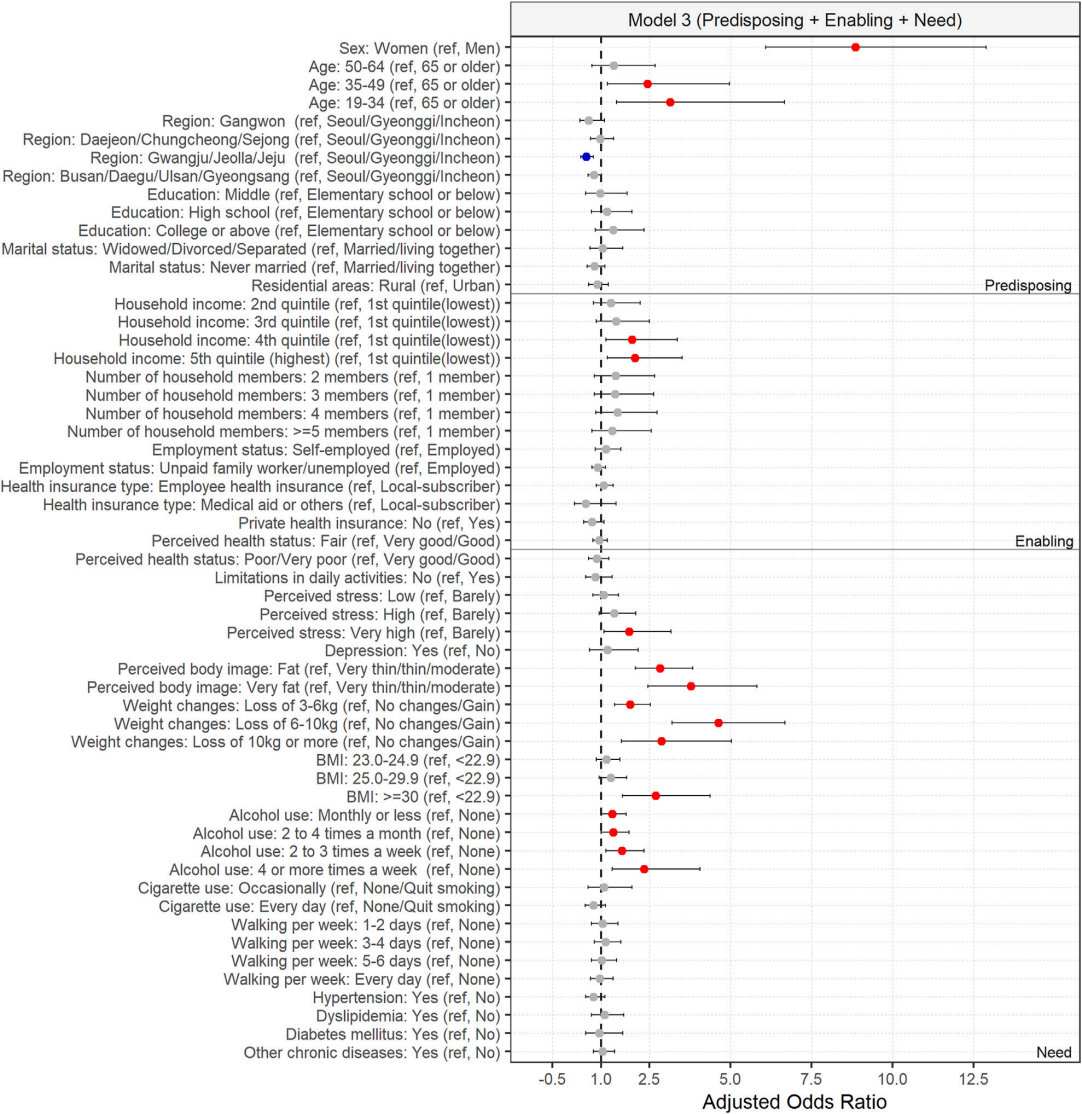
Influencing factors of herbal medicine user for weight loss in adults based on model 3.

In the fully adjusted model (model 3), women were significantly more prone to use HM for weight loss compared to men (aOR [95% CI]; 8.99 [6.18, 13.09]). Individuals aged 19 to 34 (3.35 [1.59, 7.07]) and 35 to 49 (2.57 [1.27, 5.19]) years demonstrated a higher tendency to use HM for weight loss than those aged 65 years or older. Residents of Gwangju, Jeolla or Jeju were less prone to use HM for weight loss compared to those of Seoul, Gyeonggi or Incheon (0.54 [0.38, 0.77]). Furthermore, individuals in the fourth (1.96 [1.15, 3.34]) or fifth (highest) (2.05 [1.2, 3.49]) quintile of household income were more prone to use HM for weight loss compared to individuals in the first (lowest) quintile. Individuals who experienced a very high level of stress were more prone to use HM for weight loss compared to those who barely experienced stress (1.86 [1.09, 3.18]). Individuals who observed their body image as fat (2.81 [2.06, 3.84]) or very fat (3.77 [2.45, 5.81]) were more prone to use HM for weight loss compared to those who observed their body image as very thin, thin, or moderate. Furthermore, individuals who experienced weight loss of 3–6 kg (1.88 [1.42, 2.5]), 6–10 kg (4.61 [3.19, 6.65]), or 10 kg or more (2.84 [1.61, 4.98]) in the past year demonstrated a higher tendency to use HM for weight loss compared to those with weight gain, no weight changes, or a loss of 0–3 kg during the same period. Individuals with a BMI ≥30 were more prone to use HM for weight loss compared to those with a BMI <23 (2.65 [1.63, 4.32]). Furthermore, individuals who consumed alcohol monthly or less (1.35 [1.02, 1.78]), two to three times a week (1.64 [1.14, 2.34]), or four or more times a week (2.32 [1.33, 4.04]) in the past year tends to use HM for weight loss compared to those who did not consume alcohol during the same period (Figure 2; Table 2).

**TABLE 2:** Factors of herbal medicine users for weight loss with predisposing, enabling, and need factors.

Variables	Crude analysis		Model 1		Model 2		Model 3	
	cOR (95% CI)	*p*-value	aOR (95% CI)	*p*-value	aOR (95% CI)	*p*-value	aOR (95% CI)	*p*-value
Predisposing factors								
Sex								
Men	1 [Reference]		1 [Reference]		1 [Reference]		1 [Reference]	
Women	6.23 (4.48, 8.67)	<.001	6.33 (4.55, 8.82)	<.001	6.59 (4.72, 9.19)	<.001	8.86 (6.08, 12.91)	<.001
Age (years)								
65 or older	1 [Reference]		1 [Reference]		1 [Reference]		1 [Reference]	
50-64	2.38 (1.33, 4.28)	.004	2.14 (1.14, 4.02)	.017	1.61 (0.83, 3.09)	.156	1.39 (0.72, 2.67)	.325
35-49	5 (2.87, 8.72)	<.001	4.11 (2.17, 7.77)	<.001	3.18 (1.6, 6.3)	.001	2.44 (1.19, 4.97)	.015
19-34	5.93 (3.39, 10.38)	<.001	5.6 (2.88, 10.89)	<.001	4.55 (2.24, 9.24)	<.001	3.14 (1.48, 6.66)	.003
Region								
Seoul/Gyeonggi/Incheon	1 [Reference]		1 [Reference]		1 [Reference]		1 [Reference]	
Gangwon	0.6 (0.34, 1.05)	.071	0.67 (0.38, 1.18)	.165	0.68 (0.38, 1.21)	.190	0.61 (0.34, 1.1)	.100
Daejeon/Chungcheong/Sejong	0.97 (0.68, 1.36)	.840	1 (0.7, 1.42)	.990	1.04 (0.73, 1.47)	.834	0.97 (0.67, 1.39)	.855
Gwangju/Jeolla/Jeju	0.54 (0.38, 0.76)	<.001	0.55 (0.39, 0.78)	.001	0.56 (0.39, 0.79)	.001	0.54 (0.38, 0.77)	.001
Busan/Daegu/Ulsan/Gyeongsang	0.76 (0.59, 0.98)	.034	0.79 (0.61, 1.01)	.063	0.8 (0.62, 1.04)	.093	0.78 (0.6, 1.02)	.067
Education								
Elementary school or below	1 [Reference]		1 [Reference]		1 [Reference]		1 [Reference]	
Middle	1.32 (0.73, 2.41)	.360	1.06 (0.57, 1.98)	.848	0.96 (0.52, 1.79)	.909	0.97 (0.52, 1.81)	.928
High school	2.18 (1.42, 3.35)	<.001	1.23 (0.74, 2.05)	.416	1.04 (0.62, 1.72)	.891	1.18 (0.71, 1.96)	.523
College or above	2.51 (1.65, 3.82)	<.001	1.39 (0.84, 2.31)	.197	1.07 (0.63, 1.8)	.805	1.38 (0.82, 2.33)	.231
Marital status								
Married/living together	1 [Reference]		1 [Reference]		1 [Reference]		1 [Reference]	
Widowed/Divorced/Separated	0.67 (0.45, 1.01)	.055	0.89 (0.58, 1.39)	.618	1.1 (0.69, 1.75)	.685	1.05 (0.66, 1.67)	.842
Never married	1.09 (0.86, 1.38)	.467	0.74 (0.54, 1.01)	.055	0.77 (0.56, 1.08)	.126	0.8 (0.57, 1.12)	.199
Enabling factors								
Residential areas								
Urban	1 [Reference]				1 [Reference]		1 [Reference]	
Rural	0.76 (0.55, 1.05)	.096			0.91 (0.65, 1.27)	.586	0.88 (0.62, 1.23)	.449
Household income								
1st quintile(lowest)	1 [Reference]				1 [Reference]		1 [Reference]	
2nd quintile	1.91 (1.19, 3.06)	.007			1.27 (0.75, 2.13)	.375	1.3 (0.76, 2.21)	.343
3rd quintile	2.25 (1.41, 3.6)	.001			1.39 (0.82, 2.35)	.219	1.47 (0.86, 2.5)	.161
4th quintile	2.79 (1.78, 4.37)	<.001			1.74 (1.03, 2.93)	.039	1.96 (1.15, 3.35)	.013
5th quintile (highest)	2.65 (1.7, 4.12)	<.001			1.69 (1, 2.86)	.048	2.05 (1.2, 3.5)	.008
Number of household members								
1 member	1 [Reference]				1 [Reference]		1 [Reference]	
2 members	1.67 (0.95, 2.94)	.076			1.45 (0.8, 2.62)	.217	1.45 (0.79, 2.66)	.231
3 members	2.2 (1.27, 3.78)	.005			1.43 (0.8, 2.55)	.227	1.44 (0.79, 2.62)	.229
4 members	2.61 (1.53, 4.45)	<.001			1.55 (0.87, 2.77)	.139	1.51 (0.84, 2.73)	.170
≥5 members	2.24 (1.26, 3.97)	.006			1.35 (0.73, 2.49)	.334	1.35 (0.72, 2.55)	.349
Employment status								
Employed	1 [Reference]				1 [Reference]		1 [Reference]	
Self-employed	0.8 (0.58, 1.09)	.155			1.21 (0.87, 1.7)	.263	1.15 (0.82, 1.62)	.415
Unpaid family worker/unemployed	1.03 (0.84, 1.27)	.775			0.92 (0.74, 1.14)	.429	0.9 (0.72, 1.13)	.384
Health insurance type								
Local-subscriber	1 [Reference]				1 [Reference]		1 [Reference]	
Employee health insurance	1.17 (0.95, 1.44)	.141			1.05 (0.83, 1.32)	.704	1.08 (0.85, 1.38)	.512
Medical aid or others	0.41 (0.16, 1.05)	.063			0.69 (0.24, 1.92)	.473	0.52 (0.19, 1.46)	.214
Private health insurance								
Yes	1 [Reference]				1 [Reference]		1 [Reference]	
No	0.44 (0.31, 0.64)	<.001			0.69 (0.46, 1.04)	.075	0.72 (0.47, 1.09)	.121
Need Factors								
Perceived health status								
Very good/Good	1 [Reference]						1 [Reference]	
Fair	1.05 (0.85, 1.31)	.642					0.94 (0.75, 1.19)	.626
Poor/Very poor	1.16 (0.87, 1.57)	.313					0.87 (0.62, 1.24)	.452
Limitations in daily activities								
Yes	1 [Reference]						1 [Reference]	
No	1.13 (0.73, 1.76)	.582					0.83 (0.52, 1.34)	.445
Perceived stress								
Barely	1 [Reference]						1 [Reference]	
Low	1.3 (0.91, 1.87)	.152					1.08 (0.75, 1.54)	.690
High	2.06 (1.42, 3)	<.001					1.4 (0.95, 2.08)	.092
Very high	3.18 (1.96, 5.15)	<.001					1.86 (1.09, 3.17)	.024
Depression								
No	1 [Reference]						1 [Reference]	
Yes	1.49 (0.86, 2.56)	.154					1.19 (0.65, 2.15)	.575
Perceived body image								
Gain/No changes/Loss of 0–3 kg	1 [Reference]						1 [Reference]	
Fat	2.37 (1.84, 3.06)	<.001					2.82 (2.06, 3.84)	<.001
Very fat	4.93 (3.73, 6.52)	<.001					3.78 (2.45, 5.82)	<.001
Weight changes								
No changes/Gain	1 [Reference]						1 [Reference]	
Loss of 3–6 kg	1.4 (1.06, 1.85)	.016					1.89 (1.42, 2.52)	<.001
Loss of 6–10 kg	3.48 (2.47, 4.89)	<.001					4.62 (3.2, 6.68)	<.001
Loss of 10 kg or more	2.65 (1.58, 4.45)	<.001					2.86 (1.63, 5.03)	<.001
BMI (kg/m^2^)								
<23	1 [Reference]						1 [Reference]	
23.0-24.9	0.91 (0.69, 1.19)	.472					1.17 (0.86, 1.58)	.326
25.0-29.9	0.89 (0.7, 1.13)	.344					1.3 (0.94, 1.79)	.113
≥30	2.36 (1.74, 3.22)	<.001					2.69 (1.66, 4.37)	<.001
Alcohol use								
None	1 [Reference]						1 [Reference]	
Monthly or less	1.66 (1.26, 2.18)	<.001					1.35 (1.02, 1.78)	.035
2 to 4 times a month	1.34 (1, 1.8)	.051					1.37 (1, 1.87)	.050
2 to 3 times a week	1.21 (0.87, 1.69)	.259					1.64 (1.15, 2.33)	.007
4 or more times a week	1.31 (0.81, 2.14)	.271					2.33 (1.34, 4.06)	.003
Cigarette use								
None/Quit smoking	1 [Reference]						1 [Reference]	
Occasionally	1.06 (0.63, 1.78)	.830					1.09 (0.6, 1.95)	.782
Every day	0.48 (0.33, 0.68)	<.001					0.76 (0.51, 1.14)	.190
Walking per week								
None	1 [Reference]						1 [Reference]	
1–2 days	1.19 (0.82, 1.71)	.353					1.04 (0.71, 1.52)	.852
3–4 days	1.22 (0.87, 1.72)	.255					1.13 (0.79, 1.61)	.509
5–6 days	1.23 (0.87, 1.75)	.244					1.02 (0.71, 1.48)	.904
Every day	1.04 (0.75, 1.45)	.824					0.96 (0.67, 1.37)	.827
Hypertension								
No	1 [Reference]						1 [Reference]	
Yes	0.42 (0.3, 0.58)	<.001					0.77 (0.52, 1.12)	.167
Dyslipidemia								
No	1 [Reference]						1 [Reference]	
Yes	0.58 (0.41, 0.82)	.002					1.1 (0.71, 1.71)	.654
Diabetes mellitus								
No	1 [Reference]						1 [Reference]	
Yes	0.5 (0.3, 0.83)	.008					0.94 (0.53, 1.68)	.834
Other chronic diseases								
No	1 [Reference]						1 [Reference]	
Yes	0.81 (0.62, 1.07)	.135					1.05 (0.77, 1.42)	.762

Abbreviations: aOR, adjusted odds ratio; BMI, body mass index; CI, confidence interval; cOR, crude odds ratio.

Crude analyses were obtained from simple logistic regression analyses for the individual characteristics of predisposing, enabling, and need factors. Model one was constructed from multiple regression analysis for predisposing factors. Model two was constructed from multiple regression analysis for predisposing and enabling factors. Model three was constructed from multiple regression analysis for predisposing, enabling, and need factors. Sample weights were used in all statistical analyses.

To assess the robustness of our findings, sensitivity analysis on the number of chronic diseases was performed by combining hypertension, dyslipidemia, diabetes mellitus, and other chronic diseases among the need factors in model 3. There was no difference in the significance of characteristics associated with HM use for weight loss compared to the results of model 3 (Supplementary Material).

## 4 Discussion

This study was the first to analyze the characteristics and influencing factors of adult HM users for weight loss regardless of BMI, using extensive RWD sources. It addresses the rising interest in obesity treatment and prevention for both health management and beauty purposes. For weight-loss purposes, HM users were predominantly women, younger, highly educated, with a higher income, and non-smokers. Additionally, HM users demonstrated a higher rate of severe obesity and more significant weight loss over the past year. They reported higher levels of stress and a perception of their body image as very fat. These characteristics of HM users for weight loss mirrored those found among children and adolescents, where parents had higher education levels, identified household economic status and stress levels were elevated. The prevalence of obesity among female students, based on BMI, was higher in HM users (Yim and Lee, 2023). However, particularly among female students, no significant difference was observed in identified body image between HM users and non-users (Yim and Lee, 2023). This characteristic was unique to adult HM users for weight loss. A study examining the characteristics of US adults attempting weight loss revealed that women, younger individuals, those from higher-income families, and the obese were more prone to make weight loss attempts (Martin et al., 2018). Our study suggests that the characteristics observed among US adults attempting weight loss were more pronounced among the HM users than among the non-users. In addition, HM users demonstrated a significantly higher proportion of individuals holding private health insurance, a finding consistent with the characteristics of Korean medicine healthcare users, including HM, for patients with functional dyspepsia (Lee et al., 2022b).

After adjusting for predisposing, enabling, and need factors based on Andersen’s behavior model, our findings indicate that women, younger individuals, those with higher household incomes, and adults reporting higher experienced stress were more prone to use HM for weight loss. In addition, adults perceiving themselves as having a fat/very fat body image and those categorized as severely obese by BMI were more prone to use HM for weight loss. Furthermore, compared to non-drinkers, alcohol drinkers, especially those consuming alcohol more than twice a week, showed a tendency to use HM for weight loss. Given that alcohol consumption is a known risk factor for obesity (Traversy and Chaput, 2015), it is plausible that severely obese individuals, who were more prone to use HM, also had significantly higher alcohol consumption. Significantly, HM users demonstrated a higher rate and amount of weight loss compared to the non-users. These findings can be interpreted in various ways from different perspectives. Given that the rate of severe obesity was higher among the HM users, it is plausible that the amount of weight loss following its attempts may have been greater, potentially resulting in more substantial weight loss compared to the non-users. In addition, previous ineffective ways of losing weight might also be a factor in choosing to use HM for weight loss. However, this was a cross-sectional survey of the frequency of alcohol consumption, weight loss methods, and weight change over the past year. The rate of alcohol consumption and weight loss in the HM users was higher than that in the non-users; however, a causal relationship cannot be confirmed owing to the nature of the cross-sectional study.

Similar to adults, overweight and obese children and adolescents were more prone to use HM for weight loss if they identified higher household economic status and stress levels (Yim and Lee, 2023). Additionally, students whose fathers had a high education level, and those who experienced depressed moods, or had two or more chronic allergic diseases showed a tendency to use HM (Yim and Lee, 2023). However, in adults, no significant difference was observed between the HM users and non-users. In addition, stress was one of the primary predictors of Korean medicine healthcare use for functional dyspepsia (Lee et al., 2022b), which aligns with our study findings. Given the association between stress and severe obesity (Koski and Naukkarinen, 2017) and considering the high rate of severe obesity among the HM users in our study, it is plausible that stress levels were also elevated among this group. In addition, since HM is recognized as a safe, effective, and easy-to-use method for stress management (Burns, 2023), adults experiencing high-stress levels may have used HM in anticipation of its additional benefits. Furthermore, users of HM for weight loss tend to be fewer in Gwangju/Jeolla/Jeju compared to Seoul/Gyeonggi/Incheon. As of 2021, statistical data on the number of Korean medicine clinics/hospitals by city and province indicate that over 50% of the total Korean medicine clinics/hospitals were located in Seoul/Gyeonggi/Incheon (Yearbook of Traditional Korean Medicine Publication Committee, 2021), suggesting a relatively higher accessibility to HM in these areas. The greater use of HM for weight loss in women is a similar result to previous studies, and this gender difference might be due to women being better at monitoring their health status and managing their awareness of their health (Liang et al., 1999; Stjernberg et al., 2006). In addition, increasing interest in appearance among younger adults, whose main motivation for losing weight is appearance and social factors (LaRose et al., 2013), might have resulted in the increased use of HM for weight loss.

Currently, obesity is considered a pandemic owing to its high prevalence and enormous socioeconomic burden (Tremmel et al., 2017; World Health Organization, 2021; Malkin et al., 2022). The risk associated with side effects and the high cost of pharmacotherapy, coupled with low adherence to lifestyle modifications, is driving the demand for effective and safe alternative treatment strategies, such as HM (Eldalo et al., 2017; Lewis, 2019; Cheon and Jang, 2021; Tak and Lee, 2021). Consequently, obesity poses a significant health burden. However, the findings of this study revealed significant differences in socioeconomic characteristics between the group using HM for weight loss in Korea and the group that did not. These differences were primarily associated with education level, income level, and possession of private health insurance. This suggests that individuals facing challenges with medical expenses, labor, and socioeconomic losses owing to obesity may not be opting for safe and effective HM treatment. Given the recent increase in policy and decision-making using RWD (Cave et al., 2019; Kc et al., 2023), examining the characteristics and influencing factors of HM users for weight loss based on large-scale RWD can help make policy decisions for effective resource allocation to address unmet needs for conventional treatment of obesity. In addition, healthcare providers can treat patients by considering these characteristics and needs, while researchers can use this information as a reference for designing future studies. For example, while the effectiveness and safety of HM for weight loss could not be directly compared with other treatment methods through KNHANES analysis, numerous studies have reported the effectiveness and safety of HM in this regard (Maunder et al., 2020; Park et al., 2022). However, owing to low methodological quality and poor reporting of HM in previous studies (Maunder et al., 2020; Park et al., 2022), further rigorously designed high-quality clinical trials are needed to evaluate its effectiveness and long-term safety. Furthermore, ongoing research on the HM registry for weight loss (Ko et al., 2022) holds promise for confirming not only the long-term safety and effectiveness of HM but also elucidating the characteristics of HM users and factors influencing clinical outcomes through prospective longitudinal studies. Consequently, the results of this large-scale retrospective study based on RWD may serve as a valuable reference for establishing meaningful variables and subgroups when analyzing the outcomes of prospective studies.

This study had some limitations. First, while there is generally a lower proportion of men attempting to lose weight compared to women (Martin et al., 2018), the analysis included only 50 men who used HM for weight loss. Consequently, caution is warranted when interpreting the overall results pertaining to men, due to the small sample size. Second, among the KNHANES items, health behaviors such as weight control methods, smoking, drinking, and physical activity are surveyed through self-administered questionnaires. While KNHANES data collection follows guidelines that include specific explanations for each item to ensure clarity and minimize recall bias, some level of bias may still be present. In addition, it is important to note that when participants indicated the use of HM for weight control, this may have encompassed not only HM prescribed by Korean medical institutions but also healthy functional foods containing herbs or over-the-counter herbal products. Unfortunately, owing to the lack of specific survey items addressing these distinctions, the study could not analyze the differences between them. In South Korea, HM refers to an herb or combination of two or more herbs used to prevent and treat diseases based on Korean medicine theory, using natural products such as plants, animals, and minerals. Therefore, the various types of HMs, methods of use, and accompanying treatments that respondents individually used, may have affected the user’s experience and contaminated the results. However, since these were not collected in the survey, detailed analysis was not possible. Finally, the KNHANES is a cross-sectional survey encompassing health behavior, chronic disease prevalence, health examination, and food and nutritional intake. Consequently, it is impossible to establish a causal relationship regarding HM use based solely on the results of this study. Currently, a core outcome set for behavioral weight management interventions in overweight and obese adults has been developed (Mackenzie et al., 2020), and a core outcome set for traditional Chinese medicine for the treatment of obesity is also being developed (COMET Initiative, 2024). Therefore, a longitudinal analysis should also be performed to identify the causal relationship and evaluate the effects based on the core outcome set.

This study adds valuable insights for the first time into the characteristics and influencing factors of adult users of HM for weight loss, utilizing extensive real-world data sources. By identifying demographic and health-related factors associated with HM use, the study contributes to evidence-based decision-making in healthcare policy and resource allocation for addressing obesity treatment and prevention. Understanding the characteristics of individuals who utilize HM for weight loss, such as higher stress levels, perceived body image concerns, and socioeconomic factors, can inform the development of personalized treatment strategies. Healthcare providers can use this information to tailor interventions and support programs to better meet the needs of patients seeking alternative weight loss therapies. The study’s findings shed light on socioeconomic disparities in HM use for weight loss, particularly regarding education level, income, and possession of private health insurance. This underscores the importance of addressing access barriers to conventional obesity treatment methods and ensuring equitable access to safe and effective alternative therapies like HM. Addressing these disparities can help mitigate the socioeconomic burden of obesity and improve overall public health outcomes.

## Data Availability

The original contributions presented in the study are included in the article/Supplementary Material, further inquiries can be directed to the corresponding author.

## Abbreviations

BMI, body mass index; HM, herbal medicine; RWD, real-world data; KNHANES, Korea National Health and Nutrition Examination Survey.
