# Supplementary material for: Real-world evidence of characteristics and factors influencing herbal medicine use for weight loss in adults
Source: Front Pharmacol. 2024 Jul 16;15:1437032. doi: 10.3389/fphar.2024.1437032 (PMC11286466; doi:10.3389/fphar.2024.1437032)
Supplement: Supplementary file 1 [file Table1.DOCX]

**Supplement 1. Sensitivity analysis in model 3**

| **Variables** | **Main analysis in model 3** | | **Variables** | **Sensitivity analysis in model 3** | |
| --- | --- | --- | --- | --- | --- |
|  | **aOR (95% CI)** | ***P* value** |  | **aOR (95% CI)** | ***P* value** |
| **Predisposing factors** |  |  | **Predisposing factors** |  |  |
| Sex |  |  | Sex |  |  |
| Men | 1 [Reference] |  | Men | 1 [Reference] |  |
| Women | 8.86 (6.08, 12.91) | <.001 | Women | 8.99 (6.18, 13.09) | <.001 |
| Age (years) |  |  | Age (years) |  |  |
| 65 or older | 1 [Reference] |  | 65 or older | 1 [Reference] |  |
| 50-64 | 1.39 (0.72, 2.67) | .325 | 50-64 | 1.43 (0.75, 2.72) | .276 |
| 35-49 | 2.44 (1.19, 4.97) | .015 | 35-49 | 2.57 (1.27, 5.19) | .009 |
| 19-34 | 3.14 (1.48, 6.66) | .003 | 19-34 | 3.35 (1.59, 7.07) | .002 |
| Region |  |  | Region |  |  |
| Seoul/Gyeonggi/Incheon | 1 [Reference] |  | Seoul/Gyeonggi/Incheon | 1 [Reference] |  |
| Gangwon | 0.61 (0.34, 1.1) | .100 | Gangwon | 0.61 (0.34, 1.1) | .098 |
| Daejeon/Chungcheong/Sejong | 0.97 (0.67, 1.39) | .855 | Daejeon/Chungcheong/Sejong | 0.96 (0.67, 1.38) | .825 |
| Gwangju/Jeolla/Jeju | 0.54 (0.38, 0.77) | .001 | Gwangju/Jeolla/Jeju | 0.54 (0.38, 0.77) | .001 |
| Busan/Daegu/Ulsan/Gyeongsang | 0.78 (0.6, 1.02) | .067 | Busan/Daegu/Ulsan/Gyeongsang | 0.78 (0.6, 1.02) | .065 |
| Education |  |  | Education |  |  |
| Elementary school or below | 1 [Reference] |  | Elementary school or below | 1 [Reference] |  |
| Middle | 0.97 (0.52, 1.81) | .928 | Middle | 0.98 (0.53, 1.82) | .949 |
| High school | 1.18 (0.71, 1.96) | .523 | High school | 1.2 (0.72, 1.98) | .487 |
| College or above | 1.38 (0.82, 2.33) | .231 | College or above | 1.4 (0.83, 2.35) | .211 |
| Marital status |  |  | Marital status |  |  |
| Married/living together | 1 [Reference] |  | Married/living together | 1 [Reference] |  |
| Widowed/Divorced/Separated | 1.05 (0.66, 1.67) | .842 | Widowed/Divorced/Separated | 1.04 (0.65, 1.66) | .866 |
| Never married | 0.8 (0.57, 1.12) | .199 | Never married | 0.8 (0.58, 1.12) | .201 |
| **Enabling factors** |  |  | **Enabling factors** |  |  |
| Residential areas |  |  | Residential areas |  |  |
| Urban | 1 [Reference] |  | Urban | 1 [Reference] |  |
| Rural | 0.88 (0.62, 1.23) | .449 | Rural | 0.88 (0.62, 1.23) | .446 |
| Household income |  |  | Household income |  |  |
| 1st quintile(lowest) | 1 [Reference] |  | 1st quintile(lowest) | 1 [Reference] |  |
| 2nd quintile | 1.3 (0.76, 2.21) | .343 | 2nd quintile | 1.29 (0.76, 2.21) | .348 |
| 3rd quintile | 1.47 (0.86, 2.5) | .161 | 3rd quintile | 1.46 (0.85, 2.49) | .166 |
| 4th quintile | 1.96 (1.15, 3.35) | .013 | 4th quintile | 1.96 (1.15, 3.34) | .013 |
| 5th quintile (highest) | 2.05 (1.2, 3.5) | .008 | 5th quintile (highest) | 2.05 (1.2, 3.49) | .008 |
| Number of household members |  |  | Number of household members |  |  |
| 1 member | 1 [Reference] |  | 1 member | 1 [Reference] |  |
| 2 members | 1.45 (0.79, 2.66) | .231 | 2 members | 1.44 (0.79, 2.65) | .236 |
| 3 members | 1.44 (0.79, 2.62) | .229 | 3 members | 1.44 (0.79, 2.61) | .230 |
| 4 members | 1.51 (0.84, 2.73) | .170 | 4 members | 1.51 (0.83, 2.72) | .173 |
| >=5 members | 1.35 (0.72, 2.55) | .349 | >=5 members | 1.35 (0.72, 2.54) | .351 |
| Employment status |  |  | Employment status |  |  |
| Employed | 1 [Reference] |  | Employed | 1 [Reference] |  |
| Self-employed | 1.15 (0.82, 1.62) | .415 | Self-employed | 1.15 (0.82, 1.62) | .417 |
| Unpaid family worker/unemployed | 0.9 (0.72, 1.13) | .384 | Unpaid family worker/unemployed | 0.91 (0.73, 1.14) | .407 |
| Health insurance type |  |  | Health insurance type |  |  |
| Local-subscriber | 1 [Reference] |  | Local-subscriber | 1 [Reference] |  |
| Employee health insurance | 1.08 (0.85, 1.38) | .512 | Employee health insurance | 1.09 (0.85, 1.39) | .492 |
| Medical aid or others | 0.52 (0.19, 1.46) | .214 | Medical aid or others | 0.53 (0.19, 1.48) | .226 |
| Private health insurance |  |  | Private health insurance |  |  |
| Yes | 1 [Reference] |  | Yes | 1 [Reference] |  |
| No | 0.72 (0.47, 1.09) | .121 | No | 0.71 (0.47, 1.09) | .115 |
| **Need Factors** |  |  | **Need Factors** |  |  |
| Perceived health status |  |  | Perceived health status |  |  |
| Very good/Good | 1 [Reference] |  | Very good/Good | 1 [Reference] |  |
| Fair | 0.94 (0.75, 1.19) | .626 | Fair | 0.94 (0.75, 1.18) | .606 |
| Poor/Very poor | 0.87 (0.62, 1.24) | .452 | Poor/Very poor | 0.87 (0.61, 1.23) | .423 |
| Limitations in daily activities |  |  | Limitations in daily activities |  |  |
| Yes | 1 [Reference] |  | Yes | 1 [Reference] |  |
| No | 0.83 (0.52, 1.34) | .445 | No | 0.82 (0.51, 1.32) | .415 |
| Perceived stress |  |  | Perceived stress |  |  |
| Barely | 1 [Reference] |  | Barely | 1 [Reference] |  |
| Low | 1.08 (0.75, 1.54) | .690 | Low | 1.08 (0.75, 1.55) | .683 |
| High | 1.4 (0.95, 2.08) | .092 | High | 1.41 (0.95, 2.09) | .086 |
| Very high | 1.86 (1.09, 3.17) | .024 | Very high | 1.86 (1.09, 3.18) | .023 |
| Depression |  |  | Depression |  |  |
| No | 1 [Reference] |  | No | 1 [Reference] |  |
| Yes | 1.19 (0.65, 2.15) | .575 | Yes | 1.19 (0.66, 2.15) | .571 |
| Perceived body image |  |  | Perceived body image |  |  |
| Gain/No changes/Loss of 0-3kg | 1 [Reference] |  | Very thin/thin/moderate | 1 [Reference] |  |
| Fat | 2.82 (2.06, 3.84) | <.001 | Fat | 2.81 (2.06, 3.84) | <.001 |
| Very fat | 3.78 (2.45, 5.82) | <.001 | Very fat | 3.77 (2.45, 5.81) | <.001 |
| Weight changes |  |  | Weight changes |  |  |
| No changes/Gain | 1 [Reference] |  | Gain/No changes/Loss of 0-3kg | 1 [Reference] |  |
| Loss of 3-6kg | 1.89 (1.42, 2.52) | <.001 | Loss of 3-6kg | 1.88 (1.42, 2.5) | <.001 |
| Loss of 6-10kg | 4.62 (3.2, 6.68) | <.001 | Loss of 6-10kg | 4.61 (3.19, 6.65) | <.001 |
| Loss of 10kg or more | 2.86 (1.63, 5.03) | <.001 | Loss of 10kg or more | 2.84 (1.61, 4.98) | <.001 |
| BMI (kg/m2) |  |  | BMI (kg/m2) |  |  |
| <23 | 1 [Reference] |  | <23 | 1 [Reference] |  |
| 23.0-24.9 | 1.17 (0.86, 1.58) | .326 | 23.0-24.9 | 1.17 (0.86, 1.58) | .317 |
| 25.0-29.9 | 1.3 (0.94, 1.79) | .113 | 25.0-29.9 | 1.29 (0.93, 1.78) | .122 |
| >=30 | 2.69 (1.66, 4.37) | <.001 | >=30 | 2.65 (1.63, 4.32) | <.001 |
| Alcohol use |  |  | Alcohol use |  |  |
| None | 1 [Reference] |  | None | 1 [Reference] |  |
| Monthly or less | 1.35 (1.02, 1.78) | .035 | Monthly or less | 1.35 (1.02, 1.78) | .035 |
| 2 to 4 times a month | 1.37 (1, 1.87) | .050 | 2 to 4 times a month | 1.36 (1, 1.86) | .052 |
| 2 to 3 times a week | 1.64 (1.15, 2.33) | .007 | 2 to 3 times a week | 1.64 (1.14, 2.34) | .007 |
| 4 or more times a week | 2.33 (1.34, 4.06) | .003 | 4 or more times a week | 2.32 (1.33, 4.04) | .003 |
| Cigarette use |  |  | Cigarette use |  |  |
| None/Quit smoking | 1 [Reference] |  | None/Quit smoking | 1 [Reference] |  |
| Occasionally | 1.09 (0.6, 1.95) | .782 | Occasionally | 1.09 (0.61, 1.96) | .768 |
| Every day | 0.76 (0.51, 1.14) | .190 | Every day | 0.77 (0.51, 1.15) | .197 |
| Walking per week |  |  | Walking per week |  |  |
| None | 1 [Reference] |  | None | 1 [Reference] |  |
| 1-2 days | 1.04 (0.71, 1.52) | .852 | 1-2 days | 1.04 (0.71, 1.52) | .860 |
| 3-4 days | 1.13 (0.79, 1.61) | .509 | 3-4 days | 1.13 (0.79, 1.61) | .515 |
| 5-6 days | 1.02 (0.71, 1.48) | .904 | 5-6 days | 1.02 (0.71, 1.48) | .899 |
| Every day | 0.96 (0.67, 1.37) | .827 | Every day | 0.96 (0.68, 1.37) | .829 |
| Hypertension |  |  | Number of chronic diseases |  |  |
| No | 1 [Reference] |  | 0 | 1 [Reference] |  |
| Yes | 0.77 (0.52, 1.12) | .167 | 1 | 1.03 (0.77, 1.39) | .839 |
| Dyslipidemia |  |  | 2 | 0.97 (0.59, 1.57) | .889 |
| No | 1 [Reference] |  | 3 or more | 0.92 (0.48, 1.76) | .790 |
| Yes | 1.1 (0.71, 1.71) | .654 |  |  |  |
| Diabetes mellitus |  |  |  |  |  |
| No | 1 [Reference] |  |  |  |  |
| Yes | 0.94 (0.53, 1.68) | .834 |  |  |  |
| Other chronic diseases |  |  |  |  |  |
| No | 1 [Reference] |  |  |  |  |
| Yes | 1.05 (0.77, 1.42) | .762 |  |  |  |

Abbreviations: aOR, adjusted odds ratio; BMI, Body mass index; CI, confidence interval.

Main analysis and sensitivity analysis in model 3 were performed using multiple regression analysis for predisposing, enabling, and need factors. Sample weights were used in all statistical analyses.
